# Aircraft and Airport Wastewater Surveillance for Public Health Preparedness Demands an Integrated and Sustainable Approach

**DOI:** 10.3390/v18101169

**Published:** 2026-10-21

**Authors:** Sunil R. Vaidya, Pankaj G. Pandey, Sidhartha Giri, Sanghamitra Pati

**Affiliations:** 1 ICMR-National Institute of Translational Virology and AIDS Research, Pune 411026, India; sr.vaidya@icmr.gov.in; 2 Indian Council of Medical Research, New Delhi 110029, India; sidhartha.g@icmr.gov.in; 3 ICMR-National Institute of Virology, Pune 411001, India; pankajpandey2502@gmail.com; 4 ICMR-Regional Medical Research Centre, Bhubaneswar 751023, India

**Keywords:** wastewater epidemiology, airport wastewater surveillance, aircraft wastewater surveillance, public health preparedness, polymerase chain reactions, genome sequencing

## Abstract

As domestic and international travel has expanded, the risk of the cross-border dissemination of infectious pathogens has increased, as clearly evidenced during the COVID-19 pandemic. Aircraft- and airport-based wastewater surveillance can provide timely, aggregate evidence of pathogen circulation among mobile populations and may complement clinical, genomic, and syndromic surveillance at points of entry. The strongest evidence for this approach comes from SARS-CoV-2 RNA detection and variant monitoring in aircraft lavatory wastewater, airport terminal wastewater, and airport-associated wastewater systems. However, aviation wastewater signals require cautious interpretation because detectability depends on pathogen shedding dynamics, passenger lavatory use, flight duration, sampling matrix, sample volume, assay sensitivity, sequencing depth, and the availability of epidemiological metadata. This structured narrative review synthesizes current evidence on aircraft and airport wastewater surveillance and examines the scientific determinants that influence surveillance validity and interpretation. Key methodological considerations include sampling design, sample preservation, nucleic acid extraction, molecular detection, sequencing, bioinformatics, inhibition assessment, recovery controls, assay sensitivity, and quality assurance. The review emphasizes that wastewater signals are aggregate and matrix-dependent and should not be interpreted as individual-level diagnosis, direct evidence of transmission, or definitive attribution to a specific flight, route, country, or population. Although SARS-CoV-2 surveillance is comparatively well-documented, multi-pathogen surveillance for enteric viruses, respiratory viruses, vaccine-preventable infections, emerging pathogens, and antimicrobial resistance markers requires pathogen-specific validation and clearer interpretation criteria. Aircraft and airport wastewater surveillance is therefore best viewed as an early-warning and situational-awareness approach whose value depends on converting technical signals into scientifically interpretable evidence.

## 1. Introduction

International air travel can redistribute infectious disease risks across regions before conventional surveillance systems detect local transmission. Airports function as points of entry, transit hubs, workplaces, commercial spaces, and interfaces between local and international populations. Aircraft, in contrast, represent short-duration mobile microenvironments in which passengers and crew from defined routes may contribute to a shared wastewater compartment, making aviation settings attractive for surveillance [1,2,3].

Wastewater surveillance provides a combined signal of pathogen shedding within a defined catchment. Clinical testing identifies infected individuals and supports diagnosis, treatment, isolation, contact tracing, and case-based reporting. Wastewater surveillance does not identify individuals, but it can indicate that one or more individuals within a sampled population have shed a detectable pathogen or its marker. In settings where individual testing is limited, delayed, or biased toward symptomatic disease, wastewater surveillance may provide earlier or broader situational awareness [4,5,6].

Aircraft lavatory wastewater may provide a flight-linked signal that is useful for detecting imported pathogens or variants associated with specific routes. Airport wastewater can cover a larger catchment, including travelers, airport workers, terminal facilities, sanitation systems, food-service areas, and other airport-linked activities. Aircraft wastewater is more route-specific but less sensitive to infections among passengers who do not use the lavatory. Airport wastewater is less source-specific but may better reflect sustained pathogen circulation within airport-associated populations [7,8,9,10,11,12,13,14,15,16,17,18].

The COVID-19 pandemic showed that aviation wastewater surveillance can detect SARS-CoV-2 RNA, support variant monitoring, and provide early evidence of lineage importation. Studies from multiple airport and aircraft settings have demonstrated broader feasibility of molecular detection, while genomic characterization has also been reported in selected cases [7,8,9,10,11,12,13,14,15,16,17,18]. These studies also identified important interpretive constraints, including variable sampling matrices, inconsistent metadata, differences in assay sensitivity, low or mixed viral loads, limitations in genome coverage, and uncertainty in attributing positive signals [5,19,20,21,22,23,24,25].

A consolidated scientific synthesis is necessary because the available literature remains fragmented across aircraft wastewater, airport wastewater, municipal wastewater, genomic surveillance, and travel-associated monitoring. Interpretation requires the assessment of assay performance, representativeness, metadata quality, matrix-specific limitations, and the strength of pathogen-specific evidence [1,2,3,5,16,17,20,26].

This review synthesizes evidence on aircraft and airport wastewater surveillance, identifies methodological determinants of validity and interpretability, examines the current and potential role of multi-pathogen monitoring, and highlights scientific limitations and research priorities. The review frames aviation wastewater surveillance as a complementary source of aggregate evidence whose scientific utility depends on the alignment between sampling design, laboratory methods, sequencing interpretation, and epidemiological context.

## 2. Review Scope and Approach

A narrative approach was selected as the available literature is heterogeneous. Published studies differ in surveillance objective, wastewater matrix, sampling duration, target pathogen, laboratory platform, sequencing method, metadata linkage, and interpretation strategy. The review therefore aimed to synthesize concepts, determinants, and methodological lessons.

Literature was identified through targeted searches of PubMed/MEDLINE, Scopus, Web of Science, and Google Scholar. The search strategy combined terms related to wastewater surveillance and wastewater-based epidemiology with terms related to aviation settings and pathogen detection. Search concepts included “aircraft wastewater”, “airport wastewater”, “aviation wastewater surveillance”, “aircraft lavatory wastewater”, “airport terminal wastewater”, “wastewater-based epidemiology”, “SARS-CoV-2”, “genomic surveillance”, “variant detection”, “respiratory viruses”, “enteric viruses”, “emerging pathogens”, “antimicrobial resistance”, and “public health surveillance”. Reference lists of relevant primary studies and reviews were also screened to identify additional sources.

Articles were considered relevant if they addressed aircraft lavatory wastewater surveillance, airport wastewater monitoring, travel-associated wastewater surveillance, SARS-CoV-2 or variant detection in aviation settings, multi-pathogen wastewater surveillance with relevance to airports or aircraft, or methodological determinants of wastewater detection. Studies focused exclusively on municipal wastewater were included selectively when they provided transferable evidence on assay sensitivity, molecular inhibition, sampling strategies, digital PCR, sequencing, passive sampling, or pathogen prioritization. Such evidence was interpreted as supporting context rather than direct evidence of aviation wastewater performance. The evidence strength in Table 1 and determinants in Table 2 were assigned qualitatively based on aviation-specific evidence, methodological consistency, availability of metadata, and relevance to scientific interpretation.

## 3. Evidence Base for Aircraft and Airport Wastewater Surveillance

Current evidence for aircraft and airport wastewater surveillance is concentrated on SARS-CoV-2 detection and genomic monitoring. Strong proof-of-concept evidence supports SARS-CoV-2 RNA detection in aviation-linked wastewater, while evidence for lineage and variant monitoring is moderate. In contrast, aviation-specific evidence for other viral, bacterial, and antimicrobial resistance targets remains limited [7,8,9,10,11,12,13,14,15,16,17,18,28]. Therefore, claims regarding multi-pathogen surveillance should clearly distinguish directly demonstrated evidence from plausible extensions based on municipal wastewater studies [6,13,28].

Aircraft lavatory wastewater can provide route-linked signals when infected passengers contribute detectable viral RNA and the sequencing depth is sufficient for lineage assignment [8,9,18]. Airport wastewater studies have also shown that terminal, sewer-node, and airport-associated wastewater streams can detect SARS-CoV-2 RNA and variants over time [11,13,14,15,16,17]. These findings support aviation wastewater as an additional input for genomic surveillance, particularly where clinical sequencing coverage is incomplete. However, lineage data obtained from wastewater should be interpreted as aggregate evidence. Such data can suggest that a variant is present within the sampled wastewater catchment, but they cannot identify which traveler was infected, confirm that transmission occurred during the flight, or establish the direction from which the variant was introduced unless supported by additional epidemiological information [5,20,21].

Airports receive travelers from multiple geographic origins and may capture signals from populations underrepresented in routine community testing. During COVID-19, airport wastewater monitoring detected SARS-CoV-2 circulation in airport-associated populations, and in some settings, showed trends broadly consistent with clinical surveillance [11,12,13,14,15,16,17,18]. This application is most appropriate for situational awareness rather than individual-level case detection. A positive signal should be interpreted with clinical, genomic, and epidemiological context and should not be used alone to estimate infection prevalence among passengers or attribute a pathogen signal to a specific flight, country, or group [1,2,3,16].

Aircraft wastewater and airport wastewater address different surveillance questions. Aircraft wastewater is more route-linked but is limited by small contributor numbers, variable lavatory use, restricted sample volume, chemical treatment, and short sampling windows [7,8,9,18,28]. Airport wastewater usually provides broader population coverage but lower source specificity because it may include passengers, airport staff, terminal facilities, food-service areas, cleaning activities, and connected sewer inputs [11,12,13,14,15,16,17]. Multilevel designs that compare aircraft, airport, and wider community wastewater are therefore more informative than single-matrix approaches, as they help distinguish route-associated signals from airport-level or community-level circulation [15].

Evidence for pathogens in aircraft and airport wastewaters beyond SARS-CoV-2 remains less developed. Multi-pathogen testing of aviation wastewater has shown feasibility for selected targets, and the detection of Mpox virus DNA in airport wastewater indicates that wastewater testing can be adapted when emerging pathogens become surveillance priorities [13,27,28]. Enteric viruses, including norovirus, enterovirus, rotavirus, astrovirus, sapovirus, hepatitis A virus, and hepatitis E virus, are credible targets because fecal shedding can generate detectable wastewater signals [6,28,29]. Respiratory viruses, such as influenza virus and respiratory syncytial virus, may also be detectable, but detection depends on shedding route, target stability, assay performance, and matrix-specific recovery [6,28]. Vaccine-preventable viruses and antimicrobial resistance markers may have scientific value for surveillance, but aviation-specific validation and interpretation criteria remain insufficiently developed [6,29].

Overall, the literature supports aircraft and airport wastewater surveillance as a complementary surveillance modality rather than a standalone border screening system [1,2,3,16]. Its strongest current application is early warning and genomic situational awareness for pathogens with validated assays, sufficient shedding into wastewater-relevant matrices, and clear interpretation criteria [8,9,14,15,16,17,18]. Evidence remains weaker for infection prevalence estimation, attribution of signals to defined traveler groups, and broad pathogen panels without pathogen-specific validation [6,13,28].

## 4. Methodological Lessons from COVID-19 Aviation Wastewater Studies

COVID-19 studies demonstrated the feasibility of aircraft and airport wastewater surveillance, but also showed that detection probability depends on biological shedding, access to the appropriate wastewater matrix, and assay performance [7,8,9,10,11,12,13,14,15,16,17,18,20]. Assay sensitivity alone does not determine surveillance sensitivity. Even a sensitive RT-qPCR assay cannot detect an infected passenger who does not shed into the sampled matrix, contributes below the detection threshold, or does not use the lavatory during flight [18,20].

Sensitivity and source specificity change with sampling scale. A single aircraft lavatory tank provides the most route-linked signal but is highly vulnerable to unpredictable variation. Contributor numbers may be low, particularly on short flights, and sample volume may be insufficient or diluted [7,8,9,18]. Pooled aircraft wastewater can improve detection probability across multiple flights but reduces route-level attribution. Terminal wastewater and airport sewer-node sampling increase catchment size and may improve signal detection, but they combine passengers, staff, infrastructure, and operational wastewater inputs [11,12,13,14,15,16,17]. Citywide wastewater provides broader epidemiological context but no longer represents aviation exposure alone [15]. Sampling design should therefore be guided by the surveillance objective: route-specific importation monitoring, airport-level situational awareness, or integration with community trends.

Metadata are essential for scientific interpretation. Flight origin, destination, duration, passenger load, aircraft type, lavatory configuration, servicing procedure, sample volume, time between landing and collection, storage temperature, and processing delay influence the meaning of aircraft wastewater results [8,13,17,18]. For airport wastewater, catchment definition, sewer connectivity, passenger throughput, staffing patterns, terminal operations, and weather-related dilution may be equally important [11,14,15]. Without these variables, positive signals cannot be linked to plausible exposure pathways, and negative signals cannot be evaluated for sampling adequacy.

Variant detection becomes interpretable only when sequencing quality and reporting criteria are defined. Wastewater sequencing can detect variants earlier than routine clinical sequencing in some settings, but mixed templates, low genome copy input, partial genome coverage, and amplicon drop-out may produce uncertain lineage calls [9,10,14,15,21]. Studies should report minimum genome copy thresholds for sequencing, coverage thresholds for lineage interpretation, replicate criteria, and criteria for classifying signals as detected, suspected, or inconclusive [5,20,21]. Wastewater variant reports should communicate uncertainty and should not treat lineage calls as equivalent to clinical isolate sequencing.

Positive and negative wastewater findings require cautious interpretation. A positive signal may justify closer scientific scrutiny and comparison with clinical or genomic data, but a single positive result should not be interpreted as proof of transmission, importation direction, or individual infection. Similarly, a negative result should not be used to declare an absence of infected travelers because sampling may miss low-prevalence or low-shedding events [5,20].

COVID-19 studies established technical feasibility; future research should determine how performance varies across pathogens, airports, matrices, sampling designs, and operating conditions [16,17,26].

## 5. Methodological Determinants of Validity and Interpretation

### 5.1. Sampling Matrix, Hydraulics, and Catchment Definition

The sampling matrix determines the surveillance inference. Aircraft lavatory wastewater, aircraft service-truck waste, airport terminal wastewater, sewer-node samples, and treatment-plant influent represent different catchments and should not be treated as interchangeable [7,8,9,10,11,12,13,14,15,16,17,18]. Aircraft lavatory wastewater is a narrow, time-bounded matrix that can provide route-linked evidence, but its sensitivity depends on lavatory use by infected passengers or crew and shedding of the target during flight [8,18]. Airport wastewater has a broader catchment and may improve detection probability, but it reduces source attribution because signals may originate from travelers, staff, restaurants, cleaning operations, or connected infrastructure [11,14,15].

Hydraulic conditions like wastewater flow, mixing, dilution, and retention also influence validity. Grab samples capture a single collection event and are vulnerable to temporal variation. Composite sampling may better represent airport sewer flows when infrastructure permits [5,24]. Passive sampling can extend temporal coverage and may be useful in sewer systems, but it requires the validation of adsorption kinetics, deployment duration, elution efficiency, saturation effects, and pathogen-specific recovery [24]. In aircraft lavatory systems, passive sampling is less practical because access is intermittent, and waste is removed during ground servicing. The choice of grab, composite, or passive sampling should therefore be based on the surveillance objective, flow characteristics, target pathogen, expected concentration, and sampling access.

### 5.2. Shedding Dynamics and Travel-Time Constraints

Wastewater detectability depends on shedding biology as well as infection prevalence. Fecal shedding magnitude, timing after infection, shedding duration, particle stability, and distribution between liquid and solid fractions all influence detection [4,6,28]. In aircraft wastewater, the travel window adds a further constraint. Short flights reduce the likelihood that infected individuals will use the lavatory and contribute detectable material. Longer flights increase the probability of lavatory use but may also increase dilution, chemical exposure, and nucleic acid degradation in holding tanks [8,18].

Pathogen selection should therefore be guided by documented shedding into wastewater-relevant matrices and validated assay performance. Enteric viruses are conceptually well-suited because fecal shedding directly supports wastewater detection [6,28,29]. Respiratory viruses may also be detectable, but their wastewater signals may arise from fecal shedding, saliva, respiratory secretions entering wastewater, or indirect contributions rather than the primary transmission route [6,28]. For pathogens with low or inconsistent shedding, a negative aircraft wastewater result has limited exclusion value [20].

### 5.3. Sample Concentration, Direct Extraction, and Recovery Efficiency

Sample processing should be selected according to target abundance and matrix characteristics. Concentration is not required for every surveillance target, but it becomes important when the expected genome copy numbers are low, sample volume is limited, or the assay limit of detection is insufficient for direct extraction [19,20]. Direct extraction may be suitable for high-abundance targets or rapid reporting, provided that inhibition and recovery are controlled. Concentration can improve analytical sensitivity, but it may also increase inhibitor carryover, processing time, workflow variability, and target loss during transfer steps [19,20].

Recovery efficiency varies by pathogen and processing method. Ultrafiltration, polyethylene glycol precipitation, electronegative membrane adsorption, adsorption-extraction workflows, magnetic particle capture, and Nanotrap-based methods do not recover enveloped viruses, non-enveloped viruses, bacteriophages, bacterial DNA, and free nucleic acids with equal efficiency [19,28]. A workflow optimized for SARS-CoV-2 cannot be assumed to perform adequately for norovirus, enterovirus, adenovirus, Mpox virus, or antimicrobial resistance genes [6,19,28]. Matrix-matched process controls, extraction controls, and recovery estimates are therefore required to distinguish true low-level detection from processing loss [5,20].

### 5.4. Matrix Interference and PCR Inhibition

Aviation wastewater differs chemically from many municipal wastewater matrices. Aircraft lavatory tanks may contain disinfectants, dyes, surfactants, deodorizing agents, preservatives, variable pH conditions, and high organic load, with composition varying by operator and servicing practice [19,28]. These constituents can reduce viral recovery, degrade nucleic acids, inhibit reverse transcription or PCR amplification, or interfere with extraction chemistry. Airport terminal wastewater may also contain cleaning agents, food-service waste, and variable dilution from operational water use [11,14,15].

Assay validation should therefore include matrix interference testing rather than rely only on clean-template analytical sensitivity. Essential controls include sample processing controls, extraction controls, internal amplification controls, no-template controls, positive control material, and inhibition assessment using spike-in or dilution-based approaches [5,20]. Validation should report the limit of detection in the relevant matrix, not only in nuclease-free water or buffer. Where feasible, studies should estimate the limit of blank, limit of detection, inter-run precision, intra-run precision, recovery rate, and inhibition frequency for each matrix type [20,22,23].

### 5.5. Molecular Detection Platforms: qPCR, dPCR, and Multiplex Assays

RT-qPCR remains appropriate for routine surveillance because it is widely available, scalable, and compatible with established quality-control procedures [5,20]. It determines whether a target is detected above the assay threshold. RT-dPCR may improve low-copy quantification and can be more tolerant of some inhibitors because target molecules are partitioned before amplification [22,23]. However, its limitations include cost, instrument availability, workflow complexity, and lower routine throughput in some settings. The choice between RT-qPCR and RT-dPCR should therefore be guided by the intended output: qualitative detection, trend monitoring, low-copy quantification, or confirmatory testing [22,23].

Multiplex assays can broaden pathogen coverage but require rigorous analytical validation. Primer-probe competition, differential amplification efficiency, cross-reactivity, reduced sensitivity in multiplex format, and inclusivity across genetic diversity must be assessed [6,13,28]. Out-of-sample validation is particularly important for emerging pathogens and genetically diverse viral groups. Without pathogen-specific limits of detection, cross-reactivity assessment, and interpretation rules, multiplex panels may generate technically positive results that are difficult to interpret epidemiologically [5,6,13].

### 5.6. Sequencing, Bioinformatics, and Variant Interpretation

Sequencing should be applied when the surveillance objective requires lineage assignment, variant monitoring, genomic clustering, or discovery-oriented analysis [9,10,14,15,21]. Amplicon sequencing is efficient for known targets such as SARS-CoV-2, but is affected by primer mismatch, amplicon drop-out, and uneven genome coverage [9,21]. Metagenomic sequencing can capture broader signals but generally requires higher input material, deeper sequencing, stricter contamination control, and more complex bioinformatics [6,21]. Nanopore sequencing can provide rapid turnaround, whereas short-read platforms may offer higher per-base accuracy depending on the workflow and analysis pipeline [9,21].

Wastewater sequencing should not be interpreted in the same way as sequencing a clinical isolate. Wastewater templates may contain multiple lineages, fragmented nucleic acids, low genome copy input, and environmental background nucleic acids [9,14,15,21]. Analyses require predefined minimum genome copy input, genome coverage thresholds, read-depth requirements, contamination controls, replicate criteria, and lineage-calling rules before sequencing results are interpreted. Variant abundance estimates should be reported with uncertainty, especially when mixed populations or partial genomes are present [5,21]. Bioinformatics pipelines should be documented and periodically evaluated using external reference datasets or inter-laboratory comparisons [21].

### 5.7. Analytical Detection and Epidemiological Interpretation

Analytical validity confirms that a test detects the intended target under defined conditions, whereas surveillance interpretation requires that the result is timely, matrix-appropriate, and epidemiologically contextualized [5,6,16,26]. A positive aircraft or airport wastewater result should be interpreted in relation to the sampling matrix, catchment definition, recovery efficiency, inhibition status, assay threshold, sequencing quality, and epidemiological context [5,20,21]. A negative result should be reported with appropriate limitations, particularly when contributor numbers, sample volume, or recovery controls are inadequate [8,18,20].

## 6. Beyond SARS-CoV-2: Multi-Pathogen Surveillance Potential and Evidence Gaps

The evidence base for aviation wastewater surveillance remains dominated by SARS-CoV-2, reflecting the availability of validated molecular assays, intensive clinical and genomic surveillance during the pandemic, and clear demand for variant monitoring [4,7,8,9,10,11,12,13,14,15,16,17,18]. Extension to other pathogens is scientifically plausible but requires target-specific validation. Wastewater detectability cannot be inferred from respiratory or enteric disease incidence alone; it depends on the shedding route, shedding magnitude, nucleic acid stability, environmental persistence, sample volume, matrix interference, recovery efficiency, and the intended surveillance interpretation [6,20,28].

Enteric viruses are the most direct candidates for expansion because their biology is closely aligned with wastewater detection. Norovirus, rotavirus, enteroviruses, poliovirus, astrovirus, sapovirus, hepatitis A virus, and hepatitis E virus may be shed in feces and can generate detectable wastewater signals [6,28,29]. Poliovirus has the strongest precedent because environmental surveillance is already used in eradication and importation-risk settings [29]. Norovirus and enteroviruses are also credible travel-associated targets, but aviation wastewater adds constraints that are less prominent in municipal systems, including shorter sampling windows, lower contributor numbers, inconsistent lavatory use, chemical treatment of aircraft waste, and variable waste-handling practices [18,28]. These factors reduce the exclusion value of negative aircraft wastewater results.

Respiratory viruses require a separate interpretation framework. The influenza virus, respiratory syncytial virus, rhinoviruses, adenoviruses, and other respiratory viruses may be detectable in wastewater when viral nucleic acids enter the matrix through feces, saliva, respiratory secretions, sputum, or hygiene-related waste [6,28]. Detection does not indicate wastewater-mediated transmission. For most respiratory viruses, transmission is primarily driven by respiratory droplets, aerosols, and close-contact exposure. In aviation wastewater, respiratory virus detection should therefore be reported as an aggregate shedding signal within the sampled matrix, not as evidence of transmission through wastewater or within the aircraft.

Vaccine-preventable pathogens may be relevant when wastewater detection has a clear biological and analytical basis. Poliovirus is the clearest example because environmental detection has an established scientific pattern [29]. Hepatitis A virus may also be relevant where travel-associated outbreaks and fecal shedding support environmental detection [6]. Measles virus is relevant to importation-related surveillance, but its application in aviation wastewater remains less established than poliovirus and other enteric viruses. Inclusion in wastewater panels should depend on validated assays, evidence of detectable shedding in relevant matrices, and predefined confirmatory interpretation criteria.

Emerging and re-emerging pathogens, including Mpox virus and novel respiratory or enteric viruses, may be considered when the epidemiological risk justifies targeted testing. Airport wastewater may be more suitable than aircraft wastewater for these targets because larger catchments increase detection probability [27]; however, larger catchments also reduce attribution. For antimicrobial resistance markers, airport wastewater may provide One Health and travel-associated AMR information, but interpretation is complex because resistance genes may originate from humans, food-service waste, environmental inputs, or connected sewer systems [6].

The validation gap for non-SARS-CoV-2 aviation wastewater targets remains substantial. Many candidate pathogens are supported by municipal wastewater data rather than aircraft- or airport-specific performance studies [6,28]. Before routine inclusion in aviation wastewater studies, each target requires matrix-specific recovery data, inhibition assessment, limit-of-detection estimates, inclusivity and exclusivity testing, sequencing feasibility where relevant, and predefined interpretation thresholds [5,20]. Multi-pathogen surveillance should therefore be developed through target-specific validation, beginning with pathogens for which shedding biology, assay performance, and interpretation criteria are sufficiently defined.

## 7. AI, Machine Learning, and Biosensors for Wastewater Signal Interpretation

Artificial intelligence and machine-learning methods may support aviation wastewater surveillance as analytical tools for data integration, anomaly detection, temporal trend analysis, and forecasting [30,31,32,33]. Any model used for alerting or forecasting requires out-of-sample validation across airports, time periods, pathogen targets, sampling matrices, and operating conditions [30,31,32,33].

Models trained on municipal wastewater data should not be assumed to transfer directly to aviation wastewater. Aircraft and airport matrices differ in population size, sampling frequency, contributor behavior, chemical composition, catchment definition, metadata availability, and surveillance use case [7,8,9,10,11,12,13,14,15,16,17,18,28,29]. Model performance may also be affected by missing metadata, changes in testing frequency, altered flight routes, reagent substitutions, assay drift, and seasonal travel patterns. These factors may generate false alerts or missed signals, so model outputs require validation against assay controls, inhibition status, recovery data, sequencing coverage, catchment definition, and epidemiological context [5,20,21,30,31,32,33].

Biosensors may reduce analytical turnaround time in future applications, but their role in aviation wastewater remains developmental. Evaluation should include analytical sensitivity, analytical specificity, matrix tolerance, field stability, maintenance requirements, false-positive rate, false-negative rate, and performance after exposure to lavatory chemicals or airport wastewater matrices [28,34]. Until such validation is available, biosensors should be considered adjunctive or investigational rather than replacements for PCR and sequencing workflows.

## 8. Future Research Directions

Future research should prioritize standardization and validation before expansion. Aircraft and airport wastewater surveillance requires matrix-specific methods for broader pathogen panels rather than the direct transfer of municipal wastewater protocols. Priority studies should estimate recovery efficiency, matrix inhibition, analytical sensitivity, limit of blank, limit of detection, inter-run and intra-run precision, sequencing success thresholds, and reproducibility across aviation wastewater matrices [5,19,20,21,22,23,28].

Comparative sampling studies are needed across aircraft lavatory tanks, service-truck waste, terminal wastewater, airport sewer nodes, and airport-associated influent [7,8,9,10,11,12,13,14,15,16,17,18,27,28]. These studies should report flight duration, passenger number, aircraft type, lavatory configuration, sample volume, time from landing to sampling, storage temperature, processing delay, concentration or direct extraction workflow, recovery controls, inhibition controls, genome copy thresholds, and sequencing coverage [5,20,21]. Standard reporting would improve comparability across airports and laboratories.

Multi-pathogen panels should be introduced through staged validation. Initial panels should prioritize targets with established shedding biology, scientific relevance, assay maturity, and feasible interpretation criteria [6,13,28]. Enteric viruses and poliovirus-related surveillance may be more technically suitable than many respiratory targets, although respiratory viruses may be useful when wastewater detectability has been validated [6,28,29]. Emerging pathogens should be added through targeted assays rather than broad panels without interpretation criteria [6,27].

Inter-laboratory studies using blinded proficiency panels, matrix-spiked samples, external quality assessment, and shared reference materials are needed to assess reproducibility [5,20]. Such studies should include both positive and inhibition-prone samples because false-negative risk remains a central concern in aviation wastewater matrices [20,21,22,23,24,25,26,27,28].

## 9. Conclusions

Aircraft and airport wastewater surveillance provides a complementary source of aggregate virological evidence at travel interfaces. Its strongest current value lies in early warning, genomic monitoring, and situational awareness, particularly when clinical or individual-level testing is incomplete, delayed, or unavailable. However, wastewater findings should be interpreted as population-level signals rather than direct evidence of individual infection, in-flight transmission, or attribution to a specific flight, airline, country, or community.

The scientific utility of aviation wastewater surveillance depends on the validity of the sampled matrix, the biology of pathogen shedding, the quality of sample processing, assay sensitivity, recovery efficiency, inhibition control, sequencing performance, and completeness of the epidemiological metadata. These factors determine whether a positive signal can be interpreted with confidence and whether a negative result has a meaningful exclusion value.

Overall, aircraft and airport wastewater surveillance is best understood as an interpretive surveillance tool rather than a standalone screening method. Its contribution is strongest when laboratory findings are evaluated alongside sampling context, molecular quality indicators, genomic evidence, and available epidemiological information. Clear acknowledgement of uncertainty is essential to prevent overinterpretation and ensure that aviation wastewater signals are used as scientifically grounded evidence within broader infectious disease surveillance.

## Figures and Tables

**Table 1:** Evidence synthesis of aircraft- and airport-associated wastewater surveillance studies.

Surveillance Application	Primary Matrix	Supported Inference	Main Interpretation Constraint
SARS-CoV-2 detection in travelers	Aircraft lavatory wastewater; selected transport-linked wastewater	Detects onboard shedding when infected travelers contribute to sampled waste [7,8]	Sensitivity depends on lavatory use, flight duration, sample volume, shedding, and assay LoD; a negative result does not exclude infected travelers [8,18,20].
Variant/lineage monitoring	Aircraft and airport wastewater	Detects variant signals when genome copy input and sequencing coverage are adequate [9,10,14,15]	Mixed templates, low coverage, amplicon drop-out, contamination, and incomplete metadata can weaken lineage assignment [5,20,21].
Airport-level surveillance	Terminal wastewater; airport sewer nodes; airport-associated influent	Captures aggregate signals from travelers, staff, terminal facilities, and airport sewer catchments [11,12,13,14,15,16,17]	Broader catchments improve detection probability but reduce source specificity; signals cannot be assigned to a specific aircraft or traveler group [11,14,15].
Point-of-entry early warning	Aircraft wastewater; airport wastewater; traveler-linked systems	May identify pathogen or variant introduction at international travel interfaces [9,10,14,16,18]	Requires adequate sampling, timely analysis, and sufficient route or catchment metadata; detection alone is insufficient for attribution [1,2,3,16].
Multi-pathogen surveillance beyond SARS-CoV-2	Aircraft and airport wastewater	Feasible for selected enteric, respiratory, and emerging pathogens [13,27,28]	Most non-SARS-CoV-2 targets require matrix-specific validation of shedding, stability, recovery, assay sensitivity, and interpretation thresholds [6,28].
Emerging pathogen detection	Mainly airport wastewater; limited aircraft-specific evidence	Airport wastewater can detect targeted threats such as Mpox when validated assays are available [27]	Positive signals require confirmatory testing and epidemiological context; interpretation is weak when clinical, travel, and catchment metadata are absent [6,27].
Travel-associated surveillance	Aircraft wastewater; airport wastewater; quarantine- or traveler-linked wastewater	Adds aggregate evidence to travel-associated pathogen monitoring [8,11,14]	Single-flight or short-duration sampling has low exclusion value; interpretation improves when aircraft, airport, clinical, genomic, and travel evidence are considered together [2,3,16].
Integration with community wastewater evidence	Aircraft, airport, and municipal wastewater systems	Helps distinguish route-linked signals from airport-level or local circulation [14,15]	Requires comparable assays, synchronized sampling, shared metadata standards, and cautious interpretation of discordant signals [14,15,16].

**Table 2:** Scientific and methodological determinants of validity and interpretation in aircraft and airport wastewater surveillance.

Domain	Determinant	Scientific Relevance	Interpretation Requirement
Sampling design	Matrix and catchment definition	Aircraft lavatory, service-truck, terminal sewer, airport sewer-node, and treatment-plant samples represent different populations and support different inferences.	Define the surveillance question before assigning meaning to the sampled matrix.
	Sampling method: grab, composite, or passive	Grab samples are time-specific and variable; composite and passive approaches improve temporal coverage but require method-specific validation.	Select and report the sampling method according to flow pattern, access, sampling duration, target pathogen, and required inference.
Target suitability	Pathogen shedding dynamics	Detectability depends on shedding route, magnitude, duration, and timing relative to travel. Low-shedding targets may be missed even with sensitive assays.	Prioritize pathogens with documented shedding into wastewater-relevant matrices and validated molecular targets.
Sample processing	Direct extraction versus concentration	Concentration may improve detection at low genome copy numbers but can increase inhibitor carryover, target loss, processing variability, and turnaround time.	Choose the processing workflow using matrix-specific limit of detection, expected target abundance, and sample volume.
	Recovery efficiency	Viral envelope status, particle size, adsorption behavior, solids partitioning, and matrix chemistry affect recovery across pathogens.	Use matrix-matched process controls and report recovery estimates where feasible.
Matrix effects	Matrix inhibition	Lavatory chemicals, dyes, disinfectants, surfactants, cleaning agents, pH variation, and organic load can inhibit extraction, reverse transcription, or amplification.	Include inhibition controls, extraction controls, internal amplification controls, and matrix-specific validation.
Analytical validity	Sensitivity and reproducibility	Assay performance differs between buffer validation and aviation wastewater matrices. False negatives may occur near the detection limit.	Report limit of blank, matrix-specific limit of detection, intra-run/inter-run precision, and acceptance criteria.
Analytical platform	RT-qPCR, RT-dPCR, and multiplex assay selection	qPCR supports routine detection; dPCR may improve low-copy quantification; multiplexing can reduce sensitivity or create cross-reactivity.	Match platform to purpose: detection, quantification, trend monitoring, or multi-pathogen screening; validate each target independently.
Genomic interpretation	Sequencing and bioinformatics	Low genome copy input, mixed lineages, amplicon drop-out, and contamination can affect variant assignment.	Define genome-copy thresholds, read-depth thresholds, coverage criteria, lineage-calling rules, and documented bioinformatics workflows.
Interpretive metadata	Flight, airport, and processing metadata	Lack of route, passenger, timing, catchment, sample-volume, storage, and processing metadata reduces interpretability.	Report flight origin/destination, duration, passenger load where available, aircraft type, sample volume, collection time, catchment definition, storage, and processing delay.
Sample integrity	Transport time and preservation	Delays, temperature excursions, and repeated freeze–thaw cycles can degrade nucleic acids and reduce detection probability.	Report transport conditions, storage temperature, processing delay, preservatives if used, and sample-quality limitations.

## Data Availability

The original contributions presented in this study are included in the article. Further inquiries can be directed to the corresponding author.

## Abbreviations

The following abbreviations are used in this manuscript:

AI	Artificial intelligence
AMR	Antimicrobial resistance
dPCR	Digital polymerase chain reaction
LoB	Limit of blank
LoD	Limit of detection
ML	Machine learning
PCR	Polymerase chain reaction
RT-dPCR	Reverse transcription-digital polymerase chain reaction
RT-qPCR	Reverse transcription-quantitative polymerase chain reaction
SARS-CoV-2	Severe acute respiratory syndrome coronavirus 2
